# Application of plant extended phenotypes to manage the agricultural microbiome belowground

**DOI:** 10.3389/frmbi.2023.1157681

**Published:** 2023-05-17

**Authors:** Alonso Favela, Martin O. Bohn, Angela D. Kent

**Affiliations:** ^1^ Department of Ecology & Evolutionary Biology, University of California in Irvine, Irvine, CA, United States; ^2^ Program of Ecology, Evolution, and Conservation Biology, University of Illinois at Urbana-Champaign, Urbana, IL, United States; ^3^ Department of Crop Sciences, University of Illinois at Urbana-Champaign, Urbana, IL, United States; ^4^ Department of Natural Resources and Environmental Sciences, University of Illinois at Urbana-Champaign, Urbana, IL, United States

**Keywords:** microbiome, sustainability, extended phenotypes, regenerative agriculture, plant breeding

## Abstract

Plants have a surprising capacity to alter their environmental conditions to create adequate niches for survival and stress tolerance. This process of environmental transformation, commonly referred to as “extended phenotypes” or “niche construction”, has historically been studied in the domain of ecology, but this is a process that is pervasive across the plant kingdom. Furthermore, research is beginning to show that plants’ extended phenotypes shape the assembly and function of closely associated microbial communities. Incorporation and understanding the role that plant-extended phenotypes play in agriculture may offer novel, bioinspired methods to manage our arable soil microbiomes. Here, we review the challenges agriculture faces, the plant extended phenotypes we know to shape the microbiome, and the potential utilization of this knowledge to improve the environmental impact of agriculture. Understanding how plant extended phenotypes shape microbial communities could be a key to creating a sustainable future with both plants and microbiomes in consideration.

## Introduction

Over the 20^th^ century, industrial agricultural systems have adapted to meet increased food demands by simplifying our agronomic management practices, increasing the amount of external inputs (fertilizers, pesticides, etc.), increasing the density of plants, and increased soil disturbance (tilling) (Galloway et al., 2008; Hallauer, 2009; Haddaway et al., 2017; Yang et al., 2021). These changes have resulted in extensive environmental degradation, increased greenhouse gas production, and harm to human health, and have consequently made agriculture a substantial contributor to climate change (Smith et al., 2008; Mora et al., 2018). Recent reports show that 52% of all fertile, food-producing soils globally are now classified as degraded, and it has been projected that continued intensive agriculture will lead to a 12% *decline* in global food production over the next 25 years (United Nations Conventions to Combat Desertification, 2015; Borrelli et al., 2017; Kopittke et al., 2019). As it stands, our current agricultural system is a major contributor to ecosystem-level impacts contributing (GHG production, nutrient runoff, etc.) to global change, and is vulnerable to the consequences of these changes (extreme weather events, etc.). Rethinking our agricultural system to be highly productive, sustainable, and resilient will require the collaboration of scientists and agriculture industry to generate solutions that will balance the needs of a growing population with the impacts of food production on local and global ecosystems.

A proposed solution to meet these challenges is to harness the functions of plant-associated soil microbial communities and incorporate them into modern agriculture (Antwis et al., 2017; Busby et al., 2017; Banerjee et al., 2019). A recent renaissance in microbial ecology, spurred by technological advances in next-generation sequencing and culturing methods, has begun to reveal the important roles that soil microbes play in plant health and productivity. These advances in understanding have led to a paradigm shift in which microbial communities are seen as functional drivers of their plant host (Bulgarelli et al., 2013; Philippot et al., 2013; Cordovez et al., 2019). Microbial assemblages can expand the genomic and metabolomic abilities of their immobile plant hosts, thus by influencing the recruitment of the rhizosphere microbiome, plants are afforded a mechanism by which they can evade stressors in their shared environment (Vandenkoornhuyse et al., 2015; Cordovez et al., 2019). Specifically, soil microorganisms have been implicated in the resistance to pathogens, amendments to plant nutrition, tolerance against drought, and resistance against plant pests (Philippot et al., 2013; Guo et al., 2016; Kwak et al., 2018; Seabloom et al., 2019). The physiological and ecological link between soil microbial communities and plants should come as little surprise, as these two systems have been interacting and coevolving since the inception of terrestrial land plants (Svistoonoff et al., 2008; Delaux and Schornack, 2021). Incorporation and expansion of a plant-microbiome perspective, with a fundamental view that the two systems are working in concert, are necessary to improve the productivity, sustainability, and resilience of agroecosystems.

Currently, these advances in our understanding of plant microbiome interactions have resulted in agro-industrial ventures focused on the production of microbial biostimulants that improve plant performance (*e.g.*, Novozymes, PivotBio, Valagro, Aphea Bio, Azotic, etc.). These industries culture, characterize, and design microorganisms that have beneficial interactions with plants. Plant growth-promoting microbes are then reintroduced back into the soil ecosystem or directly onto the plant (Kong et al., 2018; Sessitsch et al., 2019). While this approach has been shown to have considerable success in controlled greenhouse settings, these findings rarely hold in the field (Backer et al., 2018). Typically, this lack of success is attributed to the complex and context-dependent nature of agricultural soils (Hart et al., 2018; Kong et al., 2018). Microorganisms are extremely sensitive to environmental conditions. As a consequence, microbial biostimulants developed under controlled laboratory conditions can fail when introduced to the highly variable agroecosystems (Sessitsch et al., 2019). In addition, to establish in the agricultural environment, microbial biostimulants must compete with native soil microbiota and be compatible with conditions in the soil environment (Hart et al., 2018; Kong et al., 2018; Woo and Pepe, 2018). Furthermore, the biostimulant method of agricultural improvement is intractable at greater agronomic scales, as the production and development of microbial inocula is expensive, time-consuming, and not always rewarded. Significant advances in the usage of microbiome applicants are needed to bridge the gap between laboratory success and field failure.

Alternatively, we propose leveraging plant-extended phenotypes and niche construction theory in combination with genetics and crop breeding to harness plant-microbe interactions to enhance the sustainability of agroecosystems. Plant breeding is the genetic improvement of plants for human benefit. Plant breeders play a unique role in the agricultural system as they test, cross, and select traits of specific germplasm for improvement. Traits that have been successfully improved range in genetic complexity. Easily characterized phenotypic traits (*e.g.,* crop beauty, flavor, crop storage, and yield) have been the primary focus of breeders over human history (Diamond, 2002). Also, work has shown that difficult-to-measure complex polygenic traits can be successful targets of selection (Anderson et al., 2014). Some examples of context-dependent traits that breeders have improved include abiotic stress tolerance (Trethowan and Mujeeb-Kazi, 2008), pathogen resistance (Wille et al., 2019), increased tolerance to insect pests (Oxtoby and Hughes, 1989; Foyer et al., 2007) plant-soil allelopathy (Fragasso et al., 2013), and root traits (York et al., 2022). Here, we want to examine whether plant-associated microbial communities behave like the previously mentioned complex traits, whether microbiome structure and function can be classified as extended phenotypes, and whether they can be used to improve the sustainability of the agroecosystem. Understanding genetic associations governing plant-associated microbiomes will allow researchers and breeders to potentially control complex phenotypes associated with soil microbial communities and plant symbioses across a variety of environments and soil types (Oyserman et al., 2021).

The purpose of this mini-review is to explore the present knowledge relating plant genetics to the structure and function of plant microbiomes and to illustrate the viability of incorporating plant microbiome selection into agroecosystem management. We cover: 1) how and when plant genetic factors play a role in shaping the soil microbiome; 2) the mechanistic underpinnings of the plant genotype microbiome interaction and selection; 3) the link between microbiome selection and ecosystem function. After reviewing these topic areas, we will present a synthesis of the implications for managing agricultural microbiome functions through the concept of extended phenotypes.

## Plant genetic contribution to influencing the soil microbiome

### Evidence for the impact of plant species on the rhizosphere microbiome

A large body of research dating back to the early 19th century has focused on understanding how plants alter the physicochemical properties of soil surrounding the root zone, a phenomenon known as the “rhizosphere effect” (Waksman, 1927). These plant rhizosphere effects have been shown to influence the establishment of individual soil microorganisms from the environment (Neal et al., 1973; Bashan, 1986), thereby altering the composition of the soil microbial community as a whole (Bulgarelli et al., 2013; Philippot et al., 2013). Plant species from agroecosystems (Matthews et al., 2019) to natural systems (Saad et al., 2020) have the ability to alter soil microbial communities. A recent metanalysis demonstrated that bulk soil microbial communities are distinct from rhizosphere communities and that there is enrichment for Bacteroidetes, Proteobacteria, and Actinobacteria in the rhizosphere across plant species (Ling et al., 2022). Furthermore, a variety of plants ranging from citrus (Xu et al., 2018), rice (Edwards et al., 2014; Ding et al., 2019; Kim and Lee, 2020), maize (Peiffer et al., 2013; Walters et al., 2018; Favela et al., 2021), wheat (Mahoney et al., 2017), barley (Bulgarelli et al., 2015), *Arabidopsis thaliana* (Lundberg et al., 2012; Schlaeppi et al., 2014), beet (Zachow et al., 2014), lettuce (Cardinale et al., 2015), agave (Coleman-Derr et al., 2015), lotus (Zgadzaj et al., 2016), and desert grasses (Eida et al., 2018; Marasco et al., 2018) host distinct microbiome assemblages in the rhizosphere compared to bulk soil. Furthermore, evidence suggests that the strength of microbial recruitment varies immensely within and among plant species (Fitzpatrick et al., 2015; Fitzpatrick et al., 2018). A large amount of literature and recent meta-analysis across several plant species shows that plants broadly have a selective effect in the rhizosphere, yet a functional understanding of why and how plants do this is still not understood (Ling et al., 2022).

Furthermore, for many of the plant species mentioned above, research has shown that genetic distance predicts the rhizosphere microbiome assembly. Within *Poaceae* for example, plant phylogenetic differences are correlated with differential recruitment of the microbial community (Bouffaud et al., 2012; Bouffaud et al., 2014). These studies suggest that more related grasses recruit more similar microbial communities. Additionally, an in-depth analysis of plant microbiome assembly across 30 angiosperm species, which span 140 million years of evolution, shows that while plant species still have a rhizosphere microbiome effect, not all bacterial phyla respond to plant-rhizosphere selection or have a phylogenetic signal in the rhizosphere microbiome recruitment (Fitzpatrick et al., 2018). Fitzpatrick et al. (2018) also determined that specific plant traits (*e.g.* root physiology, productivity, and architecture) that are expected to shape the rhizosphere compartment, are themselves uncorrelated with host-plant phylogeny. Furthermore, it has been reported that selection on a cultivar genome can have secondary unintended impacts on how the host interacts with soil microbial communities and ecosystem processes (Favela et al., 2022). Interestingly, this work shows that plant species that recruit similar microbial communities have more robust negative soil feedbacks on each other, thereby providing a potential selective pressure against closely related species with similar root microbiomes.

### Allelic variation underlying plant microbiome assembly

Gene-level allelic differences cause substantial variation in microbiome assembly across plant germplasm. For example, knockout mutations in genes related to ATP binding transporters (Badri et al., 2008), secondary metabolite production (Huang et al., 2019), phytohormone production (Lebeis, 2014), immune system (Castrillo et al., 2017), symbiotic association (Zgadzaj et al., 2016), and host circadian clock homeostasis (Hubbard et al., 2017) have all been implicated in shifts in the rhizosphere microbiome. This is not surprising as the rhizosphere microbiome is an extremely complex quantitative trait. Many genes likely have the potential to influence the assembly of the rhizosphere microbiome.

Unlike other phenotypic traits, microbiome assembly is highly dependent on ecological processes (Agler et al., 2016; Banerjee et al., 2018). Using gene-knockout experiments, Zgadzaj et al. (2016) showed that *Lotus*-diazotroph symbiotic nodule formation additionally reshaped the rhizosphere microbial community (Zgadzaj et al., 2016). In the *Lotus* system, it appears as if symbiotic rhizobia populations act as an ecological ‘hub’ for dozens of species within the *Lotus* microbiome. Similarly, research carried out in oat has demonstrated that rhizosphere microbial establishment is sequential, structuring and promoting microbial interconnectedness (Zhalnina et al., 2018). Succession and founder effects have also been shown to play a substantial role in microbiome assembly among different plant taxa (Gupta et al., 2021). Furthermore, the presence of an individual bacterial genus within the microbial community could suppress and alter typical microbiome assembly processes and alter plant growth (Finkel et al., 2020). These ecological factors will need to be understood and incorporated to predictively select for host genetic variation that modifies root-associated microbial communities.

### Rules of genotype-driven microbial assembly

**Figure 1:**
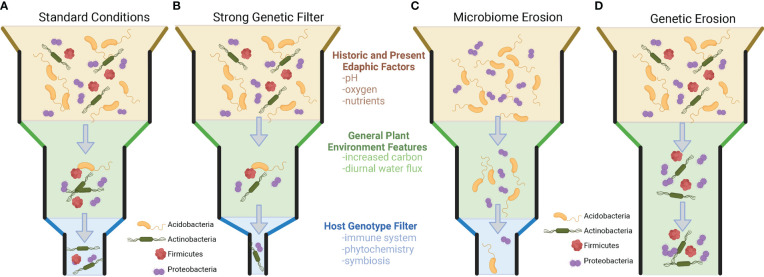
Visualization of factors in plant genotype recruitment of root microbiome from bulk soil microbial community. **(A)** The standard model of plant microbiome recruitment originally proposed in Bulgarelli et al., 2013. The original two-step selection model has been modified by the addition of an edaphic filtering effect which alters the microbial diversity present for a plant to select upon. Under the standard model, microorganisms from the bulk soil environment that interact with the rhizospheres/plant root conditions, and then finally are selected upon *via* individual host genotype differences. Panels **B-D** represent modifications on the previous model, hypothesized from the literature. **(B)** A slight modification of the model, where plant genotype selection plays a strong role in rhizosphere microbiome selection. This type of selection strongly narrows the microorganisms that are present in the rhizosphere. **(C)** In this example, edaphic factors have already reduced the diversity of the surrounding soil microbial community. While plant root and genetic filters are still present, these factors have no microbial diversity to select upon because of microbiome erosion. **(D)** A scenario where plants lack meaningful genetic variation to filter microorganisms in the rhizosphere. In scenarios **(C, D)** no plant genotype-specific microbiomes will be present.

Importantly for plant breeders, it has also been shown that within-population genetic variation exists that results in the differential recruitment of taxa to the rhizosphere, but this is not always the case. There are numerous examples where the genetic variation within and across plant species and populations does not appear to impact the recruitment of microbial taxa to the rhizosphere. Understanding when and where plant genotype plays a role in the recruitment of taxa (and the consequences for microbiome functions) will allow us to start defining how to breed for this extended phenotype (microbiome) and utilize genotype × microbiome interactions in the plant rhizosphere. [Inspired by the work of Thomas Whitman’s Community Genetics (Whitham et al., 2006; Whitham et al., 2012)]. Specifically, we propose a set of rules governing genotype effects on the community filtering of the plant microbiome. To observe a a plant genome driven microbiome, three conditions need to be present (**Figure 1**): (1) There must be genetic variation in the set of traits that are driving the microbiome (i.e., no genetic erosion, **Figure 1D**). (2) There must be sufficient microbial diversity to be shaped by the plant phenotype. (i.e., no microbiome erosion, **Figure 1C**). (3) The plant and the microbiome must be active and have a common dimension of interaction/limitation in time (i.e., shared nutrients, space, etc.). Additionally, we want to make the point that selection can decrease genetic variation in plant populations and the microbiome and can lead to genetic and microbiome erosion, which could result in the absence of a plant genotype-driven microbiome (**Figure 1**).

Studies that have reported the greatest genotype-driven rhizosphere effects are common when the analysis is either 1) conducted across a large range of environments (across the globe and continents) (Walters et al., 2018; Xu et al., 2018), thereby maximizing the microbial diversity that the genotype may select from; 2) focusing on a genetically diverse crop (*e.g.,* maize, *Arabidopsis*) (Lundberg et al., 2012; Peiffer et al., 2013; Favela et al., 2022); or 3) exploiting the extensive genetic differences existing between two cultivars (*e.g.,* wild vs. domesticated, mutant vs. wild type) (Bulgarelli et al., 2015; Pérez-Jaramillo et al., 2018; Favela et al., 2022). These three approach maximize different components of microbial recruitment. Extensive geographic analyses of rice (Edwards et al., 2014), wheat (Simonin et al., 2020), maize (Walters et al., 2018), and citrus microbiomes (Xu et al., 2018) suggest that certain microbial phylogenetic groups and specific species (OTU/ASVs) are consistently recruited (enriched in rhizosphere soils) if they are present in the starting bulk soil community prior to plant growth. While studies exist that many plant species recruit unique sets of microorganisms, evidence exists showing that this is not always the case. For example, different species of speargrass from the climatically extreme Namib desert all recruit similar plant-associated microorganisms from the surrounding soil and lack a host-specific genotype effect. This is noteworthy, as these grasses appear to vary in root traits (i.e., sheath-root system morphology) and features typically associated with differential plant microbiome community filtering (Marasco et al., 2018). Other studies have shown that successive intense selection on the microbial community through time can reduce microbial diversity and supersede previously important plant genotype community filtering on the microbiome (Morella et al., 2019). Selection, whether abiotic or biotic in origin, can erode the genetic diversity and traits of the microbial community, limiting the ability of plants to select on the community. Thereby, if microbial erosion has occurred (**Figure 1C**), genetic variation in plants that would typically alter microbiome assembly would not be observed, because of limited microbial community variation to select upon.

## Mechanistic underpinning of plant microbiome interactions

Within the rhizosphere, three genetically controlled trait classes have been described as playing a role in mechanistically shaping the microbial community: plant phytochemical allelopathy, plant immune system responses, and traits involved in symbiotic relationships with microorganisms. Here, we will cover our understanding of both the genetic and mechanistic underpinnings of microbial interactions with plants and the challenges in controlling these belowground plant traits to shape a desired outcome. Understanding the relationship of these traits to the microbiome is a critical step to enable the selection of microbiome-associated traits by plant breeders. These characteristics are important as they determine the strength and breadth of the microbiome filtering present (**Figure 1**).

### Plant chemodiversity phenotype

**Figure 2:**
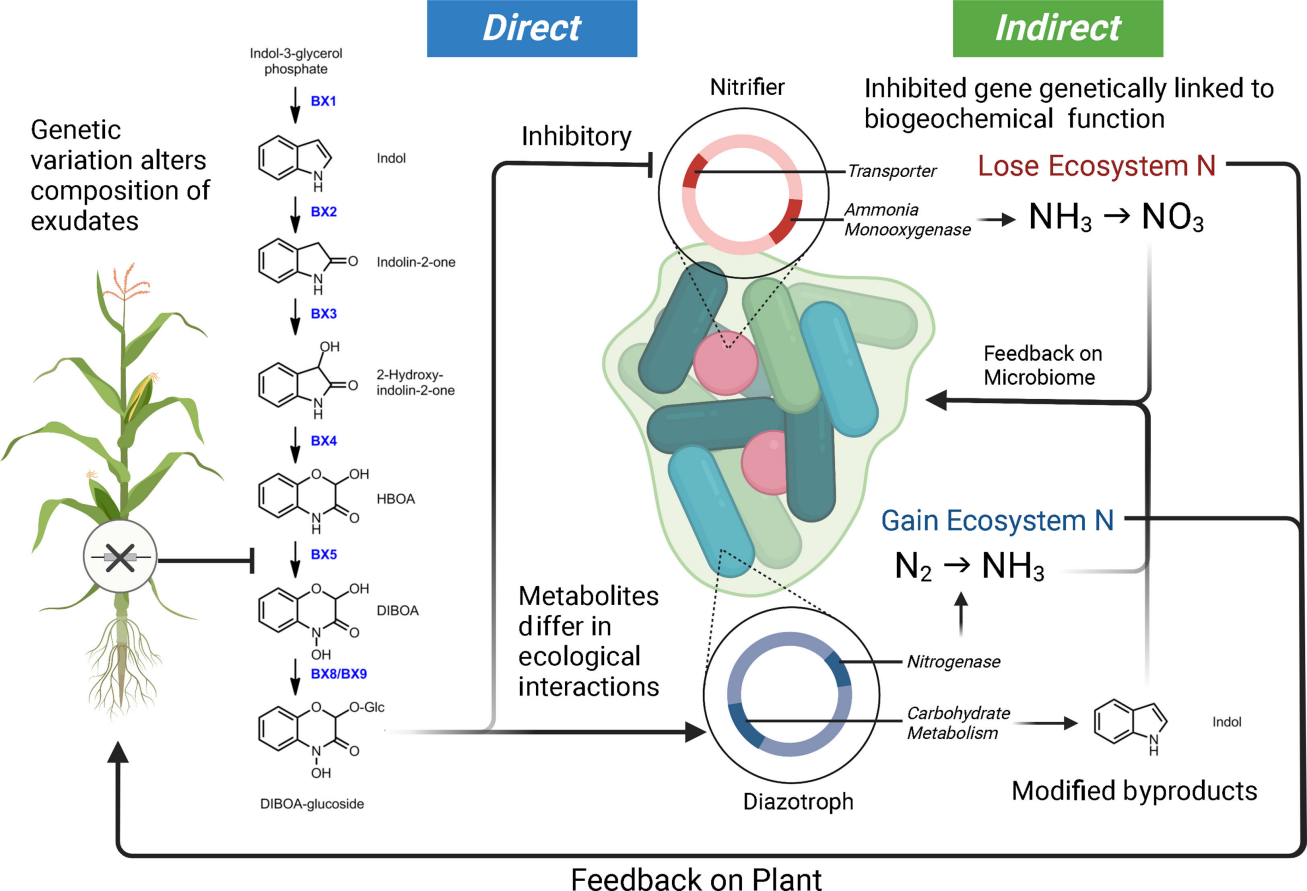
Model demonstrating how genetic variation within a maize benzoxazinoid pathway could contribute to microbiome filtering and shaping ecosystem function. In this model, we illustrate how DIBOA-glucoside (2,4-dihydroxy-2H-1,4-benzoxazin-3(4H)-one) can differentially interact with microbial taxa, consequently leading to feedbacks on microbiome composition, ecosystem processes, and plant productivity. Metabolites are from (Neal et al., 2012; Guo et al., 2016; Kudjordjie et al., 2019). The loss of maize genes upstream of DIBOA-glucoside will alter (by direction/magnitude) the sets of ecological interactions outlined here. Furthermore, the microbiome byproducts from interaction with metabolites could have indirect feedbacks on the microbiome.

The study of plant allelopathy, commonly defined as the ecological phenomena by which a plant exudes one or more metabolites to negatively influence the fitness of a competing organism, has a long history in the agricultural and ecological sciences (Cheng and Cheng, 2015; Pascale et al., 2020). Allelochemical exudates are commonly cited as playing a role in shaping the host-associated microbiota of plants (Dakora and Phillips, 2002; Van Dam and Bouwmeester, 2016; Sasse et al., 2018; Canarini et al., 2019). Plant exudates are composed of a complex mixture of carbon compounds (including organic acids, sugars, amino acids, purines, nucleosides, phenolics, and organic ions) which can be attractants or repellents to the specific microbes within the microbiome and regulate mineral acquisition chemistry (Dakora and Phillips, 2002). A considerable amount of literature makes it clear that phytochemical alterations in a single plant species will influence microbial community assembly. For example, in *Arabidopsis thaliana*, the alteration of the regulatory gene MYB72 involved in coumarin production and exudation was shown to have sweeping effects on the composition of the microbial community they established (Stringlis et al., 2018). Additionally, the coumarin scopoletin had a differential effect on various soil microbial groups, acting as an attractant for nutritional mutualists and an antimicrobial for fungi (Stringlis et al., 2018). Interestingly, studies focusing on maize and benzoxazinoid exudation have drawn similar conclusions. Genetic modifications of the plant’s phytochemical production alter rhizosphere microbial assembly (Neal et al., 2012; Hu et al., 2018; Cotton et al., 2019; Kudjordjie et al., 2019). In controlled settings, benzoxazinoids have also been shown to attract and repel different common microorganisms. A single benzoxazinoid compound can have variable effects on enriching mutualist bacteria (diazotrophs) and the suppression of antagonists (nitrifiers) (Neal et al., 2012; Otaka et al., 2022). Conceptual **Figure 2** highlights how genetic variation in a single metabolic pathway can contribute to altered microbial selection. Research is needed to characterize the full scope of ecological interactions plant chemical diversity carries out in the microbiome and niche construction (Müller and Junker, 2022).

Crop breeding for allopathic characteristics has already been proposed in previous reviews (Cheng and Cheng, 2015; Mikic and Ahmad, 2018), which outline how one would go about breeding for these characteristics. These reports described a significant amount of biochemical phenotypic variation within numerous crop cultivars and wild species (Mikic and Ahmad, 2018). This is important, as without standing genetic variation in metabolite traits, our ability to influence selection for the microbiome (and microbial functions) would be severely limited.

### Plant immune system

The plant immune system plays a critical role in shaping the microbiome, as it allows for compartmentalized and specialized responses to microbes encountered by the plant host (Jones and Dangl, 2006; Chuberre et al., 2018). Several reviews on the plant immune system have shown that roots can activate specific defense mechanisms in response to various elicitors, including molecular/pathogen-associated molecular patterns (MAMPS/PAMPS) and signal metabolites (Jones and Dangl, 2006; Chuberre et al., 2018). Further, research has shown that exposure to specific effectors can trigger plant metabolic pathways related to changes in exudate profiles (Sasse et al., 2018; Stringlis et al., 2018; Sasse et al., 2020). In many cases, the immune system-mediated responses to the microbiome are typically thought to be systemic. If a plant senses a specific effector, the phytochemistry patterns of the entire plant are altered (Korenblum et al., 2020). Plant geneticists and breeders have been able to indirectly select on the immune system (via pathogen exposure) (Vasudevan et al., 2014) for decades. Furthermore, breeding approaches for plant immune system traits are becoming more nuanced with the consideration of mutualistic microbial interactions and their ability to provide pathogen resistance and prime plant responses (Nishad et al., 2020). In summary, genetic variation in the plant immune system should strongly be considered as a target for breeding for microbiome-associated phenotypes, as it informs how a plant will respond to microorganisms.

### Plant mutualisms and symbiosis

Many agricultural plant species can form a tight symbiotic relationship with fungal and bacterial partners (Porter and Sachs, 2020; O’Brien et al., 2021). The genetic elements that underlie these phenotypes have been shown to have considerable influence on the assembly and interaction with the entire microbiome (Zgadzaj et al., 2016). This is because microbial symbioses are processes that require multiple steps of interactions, from microbial attraction *via* phytochemical production, plant immune responses that recognize the symbiotic partner, and genes involved in controlling microorganisms’ access and entrance into plant structures (Sandal et al., 2006). Research has shown that a genetic alteration to any of these elements will result in the alteration of the symbiotic rhizobacteria population, and as these can often be network hubs, genetic alterations that impact symbiotic interactions can extend to the entire microbiome (Zgadzaj et al., 2016). Furthermore, it has been shown that arbuscular mycorrhizal fungi (AMF) colonization on roots will result in altered microbiome establishment, primarily caused by extraradical hyphae association within their own distinct microbial community (Emmett et al., 2021). In addition, disrupting symbioses will also alter the biochemical profile of the plant host, which will result in further indirect effects on the microbiome (Barker et al., 2021). While covering all the interactions of plant-microbiome symbiosis are beyond the scope of this review, here we want to highlight how genetic variation in symbiotic partnership can alter the identity of the rhizosphere microbiome, by shifting keystone taxa (*e.g.,* rhizobia, AMF).

## Microbial genomes under plant selection

A functional understanding of microbial assembly should not be limited to only understanding plant characteristics. Microorganisms present in soil are immensely speciose and highly diverse with complex genomes that encode a huge array of functions, metabolites, and metabolic strategies (Torsvik, 2002; Banerjee et al., 2018; Levy et al., 2018). A large survey of 3,847 bacterial genomes revealed thousands of gene clusters that are involved in plant association (Levy et al., 2018). Functionally, genomes of bacteria that associate with plants encode more carbohydrate metabolism pathways and have a lower abundance of genetic mobile elements compared to non-plant-associated bacteria (Cole et al., 2017). Levy et al. (2018) found that across different bacterial genomes, genes clustered into units of common function. Interestingly, those functions were plant niche colonization, and microbe-microbe interactions. These results suggest that the ecological rhizosphere persistence is a driving factor in the evolution of the plant-associated microbial taxa (Levy et al., 2018).

Under the rhizosphere ecological filter model previously presented (**Figures 1**, **2**), functional genes within microorganisms will determine whether a microbe is “competent” under plant rhizosphere selection conditions (Levy et al., 2018; Oyserman et al., 2022). Connecting our understanding of bacterial genomics and plant genomics is central to providing a useful model for controlling rhizosphere microbial communities and simplifying complex ecology to a lock-and-key model (Zboralski and Filion, 2020). In this metaphor, the lock is plant mechanisms of selection (*e.g.,* phytochemistry, immune system, symbiosis) and the key is the microbial genome and functional genes. Plant mechanisms underlying microbial interaction provide a selection pressure on microbial populations in the microbiome (Oyserman et al., 2022). Well-adapted microbes will have genes to evade or benefit from plant mechanisms of selection while maintaining their primary metabolism for growth. Maladaptive microbes will lack the essential genes necessary to survive the rhizosphere selection pressure and will be unable to maintain their primary metabolism. Clearly defining the interactions between plant mechanisms of selection and the microbiome will provide a codex for directing rhizosphere and ecosystem function.

Furthermore, we attempt to highlight how plant mechanisms of selection (both direct and indirect) may be interacting with the microbial ecosystem (**Figure 2**). As mentioned above, plant inputs into the microbial ecosystem can differentially select taxa – what is critical about this plant selection is that the genetic elements in the bacterial genome under selection by the plant are in many cases physically linked to other genes (Neal et al., 2012; Kudjordjie et al., 2019; Oyserman et al., 2022). These linked genes can also carry out functional processes that can be mutualistic, antagonistic, or commensal with respect to the plant host and have the potential to alter ecosystem flux. We use a common metabolite, DIBOA-Glc, to show how microbial interactions (i.e., microbial modification to produce derivatives) with a plant metabolite can alter their subsequent interactions within the soil environment (Neal et al., 2012; Guo et al., 2016; Hu et al., 2018; Kudjordjie et al., 2019; Jacoby et al., 2020; Cadot et al., 2021). In this scenario, the exudate would inhibit the nitrifier thereby limiting nitrification, while alternatively, this same metabolite would act as a signal for *Rhizobium* and promote N-fixation (Schütz et al., 2019; Otaka et al., 2022). Indirect feedbacks from the exudation include alterations to the nitrogen environment (via nitrification and N-fixation) and accumulation of the indole byproduct. Indole was selected as the derivative form of DIBOA-Glc as it is a universal bacterial signal, which we would expect to shape microbial behavior (i.e., biofilm formation, antibiotic resistance, etc.) and play a role in microbiome assembly (Lee and Lee, 2010; Zarkan et al., 2020).

## Microbiome extended phenotype selection

We see three major complementary approaches available to breed for plant-microbiome interactions. The first approach would focus on identifying and manipulating genetic variation underpinning the extended phenotypes that control the microbiome (targeted MEPS). The second approach would focus on phenotyping microbiome function across a genetically diverse panel of lines and perform directional selection for microbiome-associated phenotypes (untargeted MEPS). The third, relatively unexplored approach, is integrating phenomics selection and spectral phenotyping as a low-cost marker of the microbiome (indicator MEPS) (Ahmadi and Bartholomé, 2022). The first scenario is ideal for well-characterized extended phenotypes like specific plant secondary metabolites (i.e., benzoxazinoids, coumarins, etc.) where the genes involved in phytochemical production and the antibiotic capacities of the phytochemical are relatively well understood. Breeding for these characteristics is relatively straightforward as marker-assisted selection and genetic manipulation can be performed on putative genetic variation. As an example, MYB72 gene-dependent coumarin production has been shown to recruit plant growth-promoting microorganisms (Stringlis et al., 2018), this gene can therefore be targeted for selection in breeding programs or be introduced into elite lines to gain beneficial microbiome-associated phenotypes. A major drawback of this known metabolic selection approach is the limitations in our current basic knowledge (alleles, compounds, pathways) involved in extended phenotypes. So far, only a few secondary plant metabolites of the thousands in existence have been characterized for their effects on soil microbial communities. More targeted work is required to determine the relative importance of chemodiversity in shaping microbial associations. A second approach to breeding for plant microbiome interactions is to phenotype the desired function of the rhizosphere microbiome – blind of plant-microbe mechanisms (untargeted MEPS). For example, if we were interested in developing lines that stimulated microbial mineralization of soil nutrients for organic agriculture, we would grow a breeding population under organic conditions and phenotype rhizosphere microbial communities collected from different host genotypes for their ability to mineralize organic nitrogen and select lines with the highest nitrogen release. After selection on these lines is carried out, plant traits can be further characterized for the causal mechanism in microbiome functional changes. The major limitation of this approach is that it is time-consuming and large enough genetic variation needs to be present in the founding population to select for microbiome differences. Furthermore, this type of untargeted MEPS needs to be done in a time-sensitive and stochasticity-aware manner, as changes in environmental conditions (*e.g.,* moisture, temperature, etc.) will alter ecosystem function and cause changes in the microbiome (potentially unrelated to host genotype). The third approach, indicator MEPS, will rely on finding spectral signals of the plants and building a relationship between this phenotype and the microbiome. This way we can quickly measure a plant trait and associate that with microbiome selection. Recent work has shown that this approach can be as predictive as genomic selection at a quarter of the cost and time (Rincent et al., 2018). Developing quick phenotypic indicators of the microbiome could allow advances in both understanding and selection for this obscured trait. Several potential approaches could be taken to breed plant-microbiome interactions into our modern agricultural system. The most straightforward method would be to select a plant trait with a known microbial/microbiome phenotype.

## Synthesis

The rhizosphere is the interface between plant roots and soil where interactions among myriad microorganisms affect biogeochemical cycling, plant growth, and tolerance to stress (Philippot et al., 2013). At this interface, we and many others have shown that plant genetics plays a role in predicting which microorganisms can grow and thrive (Lundberg et al., 2012; Walters et al., 2018; Xu et al., 2018; Favela et al., 2021; Favela et al., 2022). These differences in rhizosphere microbial diversity are important as biodiversity within the microbiome will influence ecosystem functions performed by soil microorganisms (Delgado-Baquerizo and Eldridge, 2019). To date, we have not incorporated our understanding of genotype-driven microbiome recruitment into modern agriculture. This lack of incorporation is likely because we do not understand what having a different rhizosphere microbiome means *functionally*. In this mini-review, we argue that *functional* characterization of the rhizosphere microbiome should be carried out in the context of the host extended phenotypes, and that agricultural sustainability could be improved by this incorporation.

**Figure 3:**
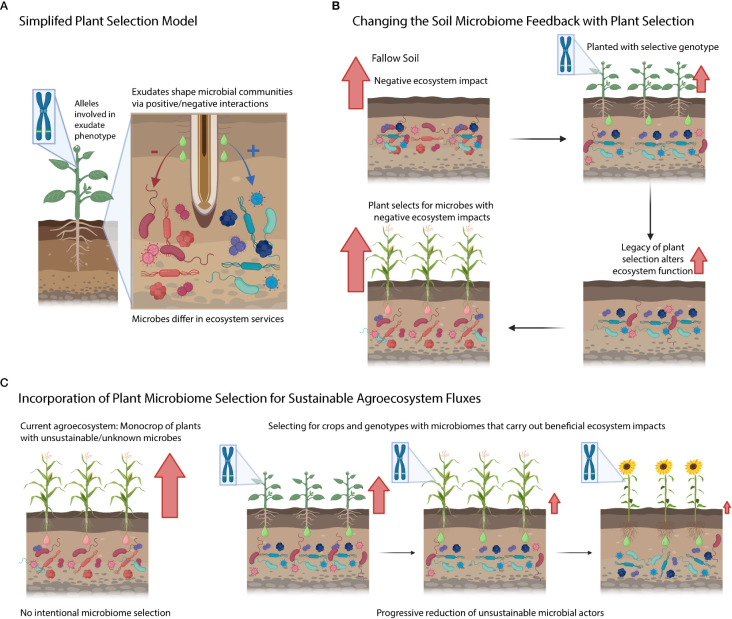
Conceptual diagram highlighting questions posed in the text. **(A)** Simplified model of plant selection. **(B)** Connection between plant microbiome selection and ecosystem processes. Red arrows denote negative ecosystem fluxes. **(C)** An idealized agricultural system where we know how genetic variation selects on soils and we intentionally grow cultivars that limit detrimental ecosystem activities mediated by the soil microbiome.

Under our simplified model, plant genotypes contain genes/phenes that selectively filter the microbiome by either leading to the enhancement or suppression of specific soil microbial taxa (**Figure 3A**). These selected taxa can be associated with ecosystem functions (*e.g.,* nitrifiers are responsible for nitrification). Yet further research is needed to determine how the rhizosphere effects scale up to the ecosystem level, and if this would considerably alter ecosystem fluxes from the agroecosystem. Additionally, research needs to be conducted to understand the legacy effects of this rhizosphere microbiome selection (**Figure 3B**) – does this plant extended phenotype of filtering soil microbiome have consequences for the next crop (potentially harming or benefiting it)? Will microbial communities under plant filtering eventually adapt/escape selection over time or will they disappear from the soil (i.e., microbial erosion)? On the plant genetics side, we are interested in understanding the key gene/phenes that should be targeted to yield the preferred microbiome (and microbial functions). Further, do these microbiome-associated traits come at a cost to yield? Finally, can we use a combination of different plant species (and genotypes) to generationally select soil microbiomes with sustainable ecosystem functions (**Figure 3C**)? Addressing these questions will enable us to improve and manage the microbiome from the genotype to the ecosystem level using plant rhizosphere selection.

Furthermore, plant extended phenotypes that shape microbiome assembly have been documented in phylogenetically diverse taxa within both monocots (Bouffaud et al., 2016) and dicots (Lundberg et al., 2012; Xu et al., 2018), leading us to conclude that rhizosphere microbiome recruitment is a fundamental function of the root and likely plays many important roles that we have just begun to characterize (Bulgarelli et al., 2013). Moreover, we know that secondary plant metabolites play a large role in controlling the microbiome (Canarini et al., 2019) and we know that evolution of plants is intimately tied to the development of novel secondary plant metabolites (Anderberg et al., 2003; Dutartre et al., 2012). Is the evolution of these secondary plant metabolites in part driven by microbiome interaction, and can rhizosphere microbiomes be predicted by broader evolutionary relatedness? Furthermore, gaining an understanding of how the diversity of plant-microbe interactions vary across the Planta kingdom may reveal novel methods to improve our agricultural system. Understanding how rhizosphere microbiomes have evolutionarily shaped plants could allow us to connect concepts from ecology, evolution, and ecosystem sciences.

Applied, this review sheds light on understudied mechanisms to alter microbial activities (by learning from plants) which could contribute to improving the sustainability of our agricultural systems (Galloway et al., 2008; Coskun et al., 2017). In theory, agronomists could pair management practices (Huffman et al., 2018) with known plant microbiome selection (*e.g.,* an organic agroecosystem paired with a crop genotype that enriches microbial mineralization) to have the germplasm work with the agricultural environment. This type of coordination between plant rhizosphere metabolic selection and agricultural fertilizer management practices could allow us to optimize the agroecosystems in a manner previously inaccessible. Yet, improving agroecosystem sustainability will require an understanding of trade-offs involved in the selection for the rhizosphere microbiome. It is possible that managing soil microbiomes through plant interactions will come at a cost to yield and will be challenging due to the complexity of microbiomes. Foundational research is needed to understand the limitations and mechanisms by which plants drive changes in soil microbiomes.

## Author contributions

AF writing—original draft preparation. AF, MB, AK contributed to writing—review and editing. All authors contributed to the article and approved the submitted version.

## Glossary

- **Extended Phenotypes (EP): **Broadly defined as all effects of a gene (or allele) on their surrounding environment (modified from (Dawkins, 1999) glossary). Furthermore, we define plant EP as traits that change microbiome recruitment and their resulting ecosystem functions. Note: We use EP and niche construction interchangeably, but in the literature, the two terms have nuance differences in ecology and evolutionary biology (Krakauer et al., 2009; Müller and Junker, 2022).
- **Niche Construction: **The process by which organisms alter environmental states, thereby modifying the conditions that they, and other organisms, experience and the sources of natural selection in their environments.
- **Community Filtering:** The abiotic and biotic conditions that select for species diversity to persist under a set of conditions. These filters may be nested, as for the rhizosphere microbiome (See **Figure 1A**).
- **Genetic Erosion:** Loss or reduction of genetic diversity between or within populations of the same species over time caused by continuous or intense selection. Genetic erosion of a plant results in the loss of extended phenotypic variation important in altering microbiome assembly (See **Figure 1D**).
- **Microbiome Erosion:** Loss or reduction of microbial diversity between or within populations of microorganisms across the microbiome caused by intense or continuous community filtering. Eroded microbiomes will lack necessary diversity to vary in response to community filtering by plant-extended phenotype (See **Figure 1C**).
- **Targeted Microbial Extended Phenotype Selection (Targeted MEPS):** Breeding approach for the EP that is informed by previous knowledge on the extended phenotypes like specific genes, pathways, or metabolites. A posterior information is needed to target specific phenotypes, highly experimentally tractable.
- **Untargeted Microbial Extended Phenotype Selection (Untargeted MEPS):** Breeding approach that relies on ecosystem functions phenotypes blind of changes to microbial composition. This approach has no clear understanding of mechanisms, is time consuming, and requires careful experiment consideration.
- **Indicator Microbial Extended Phenotype Selection (Indicator MEPS):** Breeding approach that uses a proxy plant phenotype to be predictive and indicative of changes to extended phenotypes. Requires the characterization of indication for breeding but is highly scalable if achieved.
